# DMirNet: Inferring direct microRNA-mRNA association networks

**DOI:** 10.1186/s12918-016-0373-1

**Published:** 2016-12-05

**Authors:** Minsu Lee, HyungJune Lee

**Affiliations:** Department of Computer Science and Engineering, Ewha Womans University, Seoul, South Korea

## Abstract

**Background:**

MicroRNAs (miRNAs) play important regulatory roles in the wide range of biological processes by inducing target mRNA degradation or translational repression. Based on the correlation between expression profiles of a miRNA and its target mRNA, various computational methods have previously been proposed to identify miRNA-mRNA association networks by incorporating the matched miRNA and mRNA expression profiles. However, there remain three major issues to be resolved in the conventional computation approaches for inferring miRNA-mRNA association networks from expression profiles. 1) Inferred correlations from the observed expression profiles using conventional correlation-based methods include numerous erroneous links or over-estimated edge weight due to the transitive information flow among direct associations. 2) Due to the high-dimension-low-sample-size problem on the microarray dataset, it is difficult to obtain an accurate and reliable estimate of the empirical correlations between all pairs of expression profiles. 3) Because the previously proposed computational methods usually suffer from varying performance across different datasets, a more reliable model that guarantees optimal or suboptimal performance across different datasets is highly needed.

**Results:**

In this paper, we present *DMirNet*, a new framework for identifying direct miRNA-mRNA association networks. To tackle the aforementioned issues, DMirNet incorporates 1) three direct correlation estimation methods (namely Corpcor, SPACE, Network deconvolution) to infer direct miRNA-mRNA association networks, 2) the bootstrapping method to fully utilize insufficient training expression profiles, and 3) a rank-based Ensemble aggregation to build a reliable and robust model across different datasets.

Our empirical experiments on three datasets demonstrate the combinatorial effects of necessary components in DMirNet. Additional performance comparison experiments show that DMirNet outperforms the state-of-the-art Ensemble-based model [1] which has shown the best performance across the same three datasets, with a factor of up to 1.29. Further, we identify 43 putative novel multi-cancer-related miRNA-mRNA association relationships from an inferred Top 1000 direct miRNA-mRNA association network.

**Conclusions:**

We believe that DMirNet is a promising method to identify novel direct miRNA-mRNA relations and to elucidate the direct miRNA-mRNA association networks. Since DMirNet infers direct relationships from the observed data, DMirNet can contribute to reconstructing various direct regulatory pathways, including, but not limited to, the direct miRNA-mRNA association networks.

**Electronic supplementary material:**

The online version of this article (doi:10.1186/s12918-016-0373-1) contains supplementary material, which is available to authorized users.

## Background

MicroRNAs (miRNAs) are short endogenous non-coding RNAs that regulate their target mRNAs by promoting messenger RNA (mRNA) degradation or repressing translation [2]. It has been shown that miRNAs are involved in controlling a wide range of biological processes such as differentiation [3], cellular signalling [4], and several types of cancers [2]. Since miRNAs play crucial roles in regulating genes, the functional associations between miRNAs and mRNAs should be elucidated. However, experimental identification of miRNA-mRNA associations usually performs on a small-scale with a high cost. Therefore, various computational identification methods have been proposed [5].

MiRNAs regulate their target mRNAs post-transcriptionally by base paring to complementary sequences in the 3′-UTR of mRNAs [6]. Based on this property, several methods have been proposed to identify miRNA-target mRNA relationships using sequence data based on sequence complementarity or structural stability [7–9]. Even though the sequence-based computational methods work well with generating putative miRNA-target mRNA relationships, those methods suffer from high false positive rates and false negative rates [5].

To overcome the limitation of sequence-based computational methods, matched expression profiles have been incorporated to identify miRNA-mRNA association relationships. When a miRNA regulates a target mRNA, the expression level of its target mRNA should accordingly be changed. Therefore, there is a correlation between the expression levels of a miRNA and its target mRNA. Based on the premise, various computational methods have been proposed to identify miRNA-mRNA association relationships [10–12] or to build miRNA-mRNA regulatory networks [13–16] by incorporating the matched miRNA and mRNA expression profiles. The conventional approaches for identifying miRNA-mRNA associations using expression profiles are based on traditional correlation measures such as Pearson’s linear correlation coefficient [17–19], Spearman’s rank-based correlation coefficient [20] or mutual information [21]. These conventional correlation-based methods are valuable tools for generating putative miRNA-mRNA association relationships.

However, there remain some limitations to be resolved in inferring miRNA-mRNA associations from expression data. First, traditional correlation-based network analysis results in many spurious edges [22, 23]. Most of expression profile datasets come from high-throughput experiments, and the expression profiles include hundreds to thousands of variables. The inferred correlations from the observed expression profiles using conventional correlation-based methods contain indirect association relationships derived from transitive information flow among direct associations [23]. In most cases, due to the limitations of information, it is hard to distinguish between direct associations and indirect associations among ten thousands of variables. Therefore, it is needed to suppress spurious associations from output results.

Second, the expression profiles from microarray experiments suffer from “High-dimension-low-sample-size (large p small n) problem” [24]. When we estimate the empirical correlation between all pairs of expression profiles or conditional dependencies among all variables to infer association relationships, a covariance matrix of size p × p has to be calculated. However, it is difficult to obtain an accurate and reliable estimate of the population covariance matrix from a dataset that has a large number of variables but includes few samples (n < <p) [24].

Third, it is impossible to know in advance which method will produce good results with user’s datasets among various computational methods. It has been shown that there is no single computational method that performs well consistently across different datasets and different experimental environments [25]. Each method has been developed with a different premise and approach. Thus, different computational methods usually produce different outputs from the same input data, and one method usually shows different prediction performance across different datasets. As shown in the Result section, our empirical experiments on three datasets confirm the inconsistent performance of computational methods for identifying miRNA-mRNA association relationships. Therefore, a more reliable model that guarantees optimal or suboptimal performance across different datasets is highly needed.

In this study, we present a new framework for reconstructing direct miRNA-mRNA association networks from expression data. The main objectives of the proposed framework (called DMirNet) are as follows: 1) to identify direct associations between miRNA and mRNA, 2) to handle the large p small n problem in microarray expression data, and 3) to build a reliable and robust model across different datasets. To achieve the aforementioned objectives, we propose a direct miRNA-mRNA association network reconstruction method that adopts direct correlation identification methods, the bootstrapping, and an Ensemble approach. First, to suppress indirect associations from the observed expression profiles, we adopt three methods to identify direct relationships, namely partial correlation [24], sparse partial correlation [22], and network deconvolution [23] methods. Second, to overcome the high-dimension-low-sample-size problem, we reduce the dimension of a dataset by selecting the differentially expressed miRNA and mRNAs in an experiment. Also, we embed the bootstrapping approach to build a more accurate and reproducible network by fully utilizing the limited size of samples. Third, to improve the accuracy and reliability of the inferred association relationships, we select a non-parametric Ensemble approach. It has been shown that the ensemble methods that integrate different methods usually outperform individual methods [24, 25]. To aggregate bootstrapping results and different results from different methods, we choose a rank-product-based non-parametric Ensemble method.

We use experimentally confirmed miRNA-mRNA association datasets to evaluate the performance of DMirNet. The results of our empirical experiments on three matched miRNA and mRNA expression profiles show that DMirNet reconstructs a more accurate and reliable miRNA-mRNA association network by incorporating direct correlation methods, bootstrapping and Ensemble approach. We also compare the performance of DMirNet with the state-of-the-art Ensemble model [1] that combines Pearson’s correlation, IDA [14], and Lasso [26] on the same datasets. The results of comparative experiment show that DMirNet performs better than the counterpart model with a factor of up to 1.29.

## Methods

### Framework for identifying direct miRNA-mRNA association relationships

**Fig. 1:**
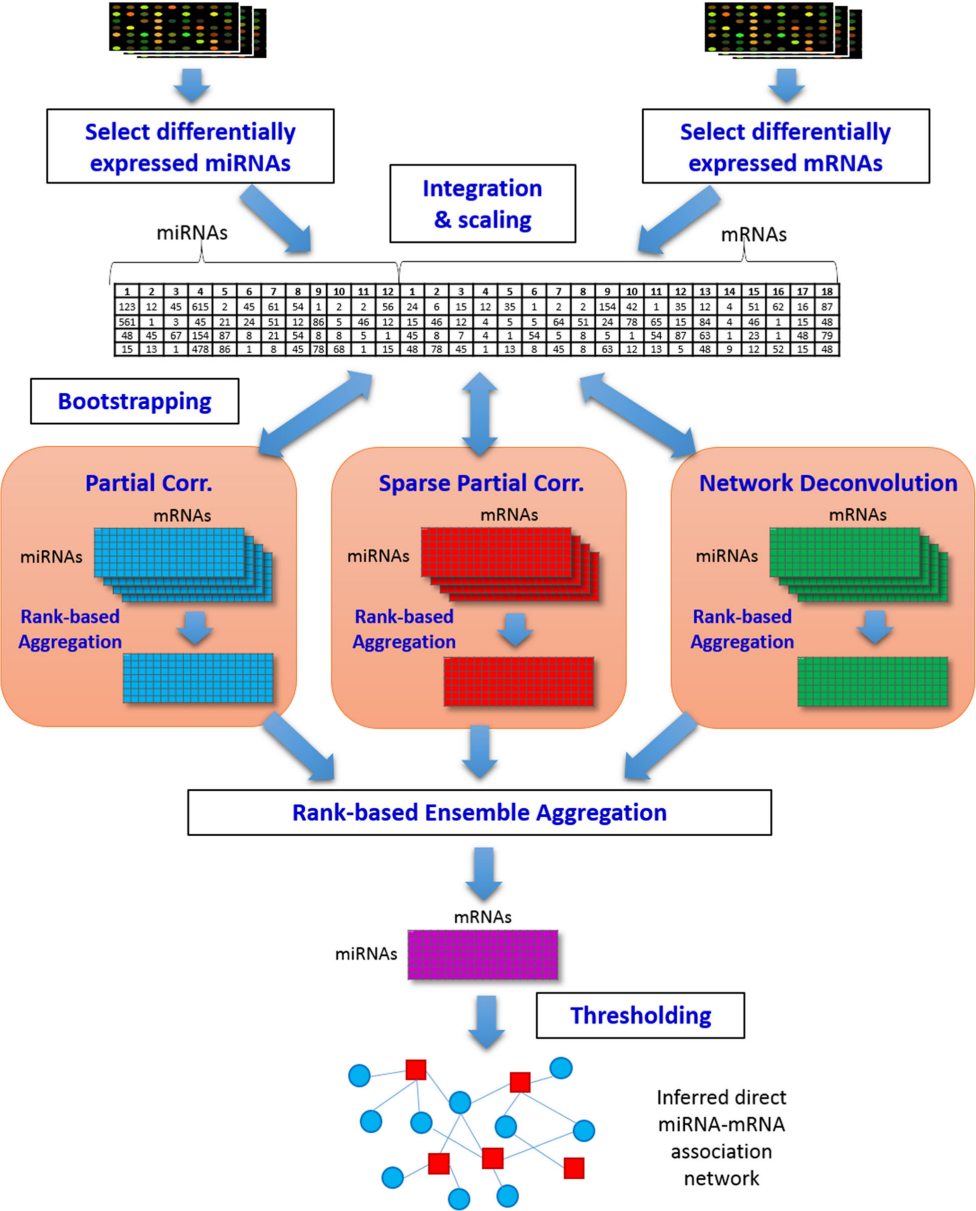
Workflow for inferring direct miRNA-mRNA association relationships

In this section, we present an overview of the framework for identifying direct miRNA-mRNA association relationships as illustrated in Fig. 1. To infer direct miRNA-mRNA association relationships, a matched miRNA-mRNA expression data is needed. After pre-processing each sample, differentially expressed miRNAs and mRNAs are identified to reduce the dimension of data and to focus on the active miRNA-mRNA associations. Because miRNA and mRNA expression profiles are obtained from different platforms, their selected miRNA and mRNA expression profiles are integrated and then scaled.

To reconstruct base-direct microRNA-mRNA association networks, three bootstrapping-based direct correlation inference methods are applied to the integrated expression profiles. Notably, each direct correlation inference method produces a direct correlation model from the expression profiles as a form of a matrix that contains all combinations among miRNAs and mRNAs. Given the integrated expression profiles, the bootstrapping generates *m* new training data sets by resampling with replacement. For each direct correlation inference methods, *m* models are computed using the generated *m* bootstrap samples that are integrated by a rank-based aggregation method. Then, the bootstrapping outputs from the three methods are integrated using the rank-based aggregation method to produce a final direct correlation model. A direct miRNA-mRNA association network is reconstructed by thresholding the weights in the output correlation matrix.

### Three direct association network inference methods

A conventional approach to reconstruct gene regulatory or association networks consists of computing the association weight among variables and inferring a link between the two variables by thresholding the association weight. However, the association weight also includes the confounding effect of other variables. By factoring out the dependency of other variables, a direct association network can be inferred. In this subsection, we introduce three methods that we have adopted for inferring direct association networks using expression profiles.

### Partial correlation

A partial correlation measures the association weight between two random variables by suppressing the effect of a set of controlling random variables. The partial correlation-based methods can infer the conditional dependency by the non-zero entries in the concentration matrix which is the inverse of covariance matrix. When we apply the partial correlation-based method to identify a genetic network, the zero entries can be interpreted as two nodes that do not interact directly with each other.

Schafer and Strimmer [24] proposed a statistically efficient and computationally fast shrinkage estimator for the covariance and correlation matrix. We use the Corpcor package [24] to compute the partial correlations between selected miRNA and mRNA expression profiles. The resulting partial correlation coefficient between the two variables is regarded as an association weight between them.

### Sparse partial correlation estimation (SPACE)

SPACE is another method to compute partial correlations under the large p and small n problem setting [22]. The main characteristics of SPACE are that it assumes that the partial correlation matrix is sparse, and most variable pairs are conditionally independent. Therefore, the output of space is a sparse matrix where many of the possible interactions are zeros. This method helps to select non-zero partial correlations. It estimates sparse partial correlation using sparse regression techniques and optimizes the results with a symmetric constraint and an *L*_1_ penalization [22].

### Network deconvolution

Network deconvolution is a direct dependency network inference method that eliminates an indirect weight from the inferred dependency network from the observed data [23]. The network deconvolution method assumes that the measured association weights from the observed data represent the sum of direct and indirect weights. Moreover, the method assumes that the indirect information flow can be approximated as the product of direct association weights. Let ***G***_*obs*_ be an observed dependency network, ***G***_*tru*_ a true direct dependency network, and ***G***_*ind*_ an indirect dependency network. Then, the indirect network can be expressed in terms of all indirect effects along paths of increasing length, and we can express the observed network (***G***_*obs*_) in terms of the true network (***G***_*tru*_) and the indirect network (***G***_*ind*_) as follows:

1 $$ {G}_{obs}={G}_{tru}+{G}_{ind}={G}_{tru}+\left({G}_{tru}^2+{G}_{tru}^3+{G}_{tru}^4+\dots \right)={G}_{tru}{\left(I-{G}_{tru}\right)}^{-1} $$

Therefore, the network deconvolution method [23] infers true direct dependency network by reversing the effect of transitive information flow across all possible indirect paths. That is, the true direct network can be calculated using the observed network as follows:

2 $$ {G}_{tru}={G}_{obs}{\left(I+{G}_{obs}\right)}^{-1} $$

The network deconvolution method can be applied with various correlation measures. In this study, we compute the pair-wise observed correlations between miRNA and mRNA expression profiles using mutual information, and then apply the network deconvolution method to suppress indirect correlation relationships from the observed correlations.

### Bootstrapping

Bootstrapping is a method for generating multiple versions of a model, and using these to generate an aggregated model. It is designed to improve accuracy and stability [27]. Given a training set *D*, bootstrapping generates *m* new training data sets *D*_*i*_ by sampling from *D* uniformly and with replacement. The *m* models are computed using the generated *m* bootstrap samples and combined by aggregating the outputs.

Because the bootstrap aggregation usually reduces variance and helps to avoid overfitting, the bootstrap procedure works well when the sample size is insufficient for straightforward model inference. Therefore, we adopt the bootstrapping procedure to reconstruct multiple networks from a single original dataset using a single direct association network inference method, which can then be aggregated into a more accurate and reproducible association network.

### Rank-based Ensemble aggregation

Because computational methods often show varying performances across different datasets [25], it is necessary to improve the reliability and accuracy of the inferred networks using computational methods. In this case, the Ensemble methods that integrate different methods can be used because they have shown better performances than individual methods [1, 25]. Also, the Ensemble methods may be useful to capture nonlinear relationships as well as linear relationships among variables by integrating results from linear or nonlinear correlation inference methods.

When several results from computational methods are integrated, the distribution of the weights between two elements usually varies considerably among computational methods. It is difficult to directly integrate real-valued weights between two variables from individual methods. Thus, it is challenging to aggregate real-valued weights of inferred association networks from different methods or datasets.

To aggregate different output networks from various methods, we adopt a non-parametric approach based on ranking. Because a rank-based Ensemble aggregation method only considers the rank of the weight and does not assume specific distribution of the source data, the rank-based method does not depend on the actual distribution of weights derived from different methods [28]. The characteristic of rank-based aggregation is the ability to combine lists from different sources and platforms. Hence, we employ a rank-based Ensemble approach to aggregate the outputs from bootstrapping iterations and different methods. The conventional rank-based aggregation methods include the rank-sum-based approach, average-rank-based approach, and Borda count election [1]. In this study, we use an inverse-rank-product method [29] to combine networks reconstructed from the same set of genes, after empirically comparing the performances of the Borda count election method and the normalized-weight-sum method with the inverse-rank-product method. The rank of a particular weight between a miRNA and an mRNA in the aggregated network is calculated by taking the product of the ranks of the same edge across all networks. Then, to assign a lower rank to a higher weight, the inverse of rank-product is used as a representative association weight between the miRNA and the mRNA. Let *G* be a set of association networks to be integrated, and let *r*_*ij*_ be a rank of association weight between node *i* and *j*. Then, the association weight of an integrated graph using the inverse-rank-product strategy (*r* ^*’*^_*ij*_) can be calculated as follows:

3 $$ {r}_{ij}^{\hbox{'}}=\frac{1}{ \log \left({\varPi}_{m\in G}\left({r}_{ij}^m+1\right)\right)} $$

We apply the inverse-rank-product method to aggregate bootstrapping outputs from the single direct association identification method and to integrate the outputs from different methods.

### Experiments for performance evaluation

To evaluate our proposed DMirNet, we performed empirical experiments with three matched miRNA and mRNA expression profiles. First, we analysed the effect of bootstrapping and Ensemble to identify miRNA-mRNA association relationships. Second, we compared the performance of DMirNet with a best-performed Ensemble model [1] for inferring miRNA-mRNA regulatory relationships from expression data.

### Experimental datasets

To avoid the biased or intentional selection of experimental data, we used the same three matched miRNA and mRNA expression profiles used in a recently published comparative study [1, 30]. The three processed datasets were obtained from [30].

Epithelial to Mesenchymal Transition (EMT) data includes the matched miRNA-mRNA expression profiles of epithelia class (11 samples) and mesenchymal class (36 samples). Multi-Class Cancer (MCC) data includes 60 samples from normal and cancerous tissues from eight organs. Breast Cancer (BR) data has 50 samples from basal and luminal groups. After applying the differentially expressed gene (DEG) analysis with *limma* package of Bioconductor and a false discovery correction process at a significant level (adjusted p-value <0.05), 35 miRNAs and 1154 mRNAs were identified as DEGs of the EMT data; additionally, 108 miRNAs and 1860 mRNAs were identified as DEGs of the MCC data. Regarding the BR data, 92 miRNAs (adjusted p-value <0.2) and 1500 mRNAs (adjusted p-value <0.0001) were identified as DEGs. The selected and integrated miRNA and mRNA expression profiles were standardized across samples before applying our DMirNet.

### Implementation of DMirNet

To identify a direct miRNA-mRNA association network, its base association networks were reconstructed using the three direct association relationships inference method with bootstrapping. For each method, the base miRNA-mRNA association networks were iteratively built using randomly resampled data with replacement. To get the bootstrapping results, we randomly selected 95% of the dataset with replacement and iteratively rebuilt association networks 100 times for each dataset.

To utilize three direct association network identification methods, we use *corpcor* and *space* R packages [31, 32] from Bioconductor and an existing network deconvolution algorithm [33]. Aggregations of the results from bootstrapping of a single method and Ensembles of different methods were performed using equation (3).

### Performance evaluation method

Currently, 1,881 miRNA precursors and 2,588 mature sequences in the Human genome are listed in miRBase (GRCh38), and the number of human genes is estimated at 20,000-25,000 [34]. Several manually curated miRNA-target mRNA databases show that one miRNA may regulate many genes as its targets, while one gene may be targeted by many miRNAs. This indicates that the relationships between miRNAs and their target mRNAs may not be one-to-one. However, the number of experimentally validated miRNA-mRNA interactions for evaluating a computational model has been very limited until now. Since there is no complete ground-truth for evaluating performances, the union of public miRNA-target mRNA databases, which include both experimentally verified relationships and some predicted relationships, has been used to evaluate performance and to compare different computational methods [1, 30, 35, 36]. The union of Tarbase v.6.0 [37], miRecords v2013 [38], miRWalk v2.0 [39] and miRTarBase v.4.5 [40] includes 62,858 unique miRNA-target mRNA interactions among 693 miRNAs and 16,091 genes. We use the union of these four databases [30] as a ground-truth dataset.

Based on the ground-truth data, the performance of each method was evaluated by checking the number of overlaps between top *k* high-ranked mRNAs of each miRNA on an inferred network and the ground-truth miRNA-mRNA pairs. Even though the number of ground-truth is very limited, the fraction of inferred correlations that are experimentally validated pairs may be regarded as a measure of the precision of the computational method. Since the total number of selected miRNA-mRNA correlations is same across all the methods in the comparative study, a higher number of overlaps can be regarded as higher precision on inferring direct miRNA-mRNA association network.

## Results

### Performance evaluation of DMirNet

To investigate the performance of DMirNet and to examine the effects of all components of the framework, we performed comparative empirical experiments using EMT, MCC, and BR datasets and three direct correlation inference methods: Corpcor, SPACE, and mutual information-based network deconvolution (MIND). For bootstrapping execution, the number of bootstrapping iterations was set to 100, and the sampling rate was set to 95%. Additionally, an inverse-rank-product method was applied for aggregating bootstrapping results and integrating results from different methods. For each method, the number of experimentally confirmed miRNA-mRNA associations was evaluated as a measure of precision by computing the overlaps between ground-truth pairs and inferred top 100 mRNAs per a miRNA. Table 1 summarizes the precisions of all combinations of DMirNet component.

**Table 1:** Number of experimentally confirmed miRNA-mRNA associations by the ground-truth data

		Single Method			Ensemble Method			
		Corpcor	Space	MIND	C&S	C&M	S&M	C&S&M
EMT	Whole	35	45	24	45	34	35	41
	Bootstrap	32	38	25	40	24	37	40
MCC	Whole	200	183	210	204	206	201	209
	Bootstrap	211	204	207	201	217	220	216
BR	Whole	98	83	95	90	94	97	102
	Bootstrap	107	95	99	99	102	100	105

The Top 100 correlations for each miRNA were selected from each experiment for performance comparison. To evaluate the effect of three direct correlation inference methods, bootstrapping and Ensemble approach, we performed a comparative study using EMT, MCC and BR datasets. Corpcor (denoted as C) is the partial correlation estimation method, SPACE (denoted as S) is the sparse partial correlation estimation method, and MIND (denoted as M) is the mutual information-based network deconvolution method. ‘Whole’ means that the whole expression profiles were used to infer a direct correlation matrix, and ‘Bootstrap’ means that 100 direct correlation matrices were computed using 100 bootstrapped samples and then aggregated based on an inverse-rank-product method

First, we investigated each single direct correlation estimation method across three datasets. The results of empirical experiments confirm that there is no single inference method that performs optimally across all datasets. Corpcor (C) shows the best precision with the BR dataset, but it ranks the medium with the EMT and the MCC datasets. SPACE (S) performs best with the EMT dataset, but has the worst performance with BR and MCC datasets. On the other hand, even though MIND (M) performs worst with the EMT dataset, it shows good performance with both MCC and BR dataset. The results indicate that each method has its own limitation on inferring direct correlations; thus, it is difficult to identify the whole direct miRNA-mRNA correlations using any single method. In such cases, the Ensemble aggregation of different methods can improve the accuracy and stability of an inferred correlation network.

We also determined the effects of bootstrapping in DMirNet framework. By applying a bootstrapping strategy, the precision of three methods was strictly increased within MCC and BR datasets. However, regarding the EMT dataset, bootstrapping does not lead to any performance improvement. The results imply that the bootstrapping procedure does not guarantee an increase in the fraction of experimentally validated pairs among inferred pairs.

Although an Ensemble method that combines three inference methods (C&S&M) shows good performance, on occasion, single methods (SPACE with EMT whole and Corpcor with BR bootstrap) or Ensembles of two inference methods (S&M with MCC bootstrap) outperforms C&S&M. This phenomenon was derived by combining the worst-performed model to the Ensemble. For example, MIND shows the worst performance with the EMT dataset but the Ensemble method excluding MIND (i.e. C&S) with the EMT dataset performs best. It should be noted that although C&M, S&M, and C&S&M perform relatively worse because they are integrated with MIND, the combined ensemble models turn out to outperform MIND itself. Additionally, when the number of aggregated methods increases from two to three, the precision of Ensemble methods also increases. The experimental results show that the Ensemble aggregation approach helps to relieve the effect of the worst model and achieves a relatively higher performance.

We also investigated the combinatorial effect of bootstrapping and Ensemble aggregation on DMirNet framework. Regarding the EMT dataset, there was no improvement in the precision using bootstrapping. However, the Ensemble aggregation of different methods reduced the effect of the worst-performed MIND. In the MCC and BR dataset, the results show performance improvements by bootstrapping across almost all experiments, as well as a relief of the effect of the worst model (SPACE) and improved precision by Ensemble aggregation. Regarding the BR dataset, each method with the combination of bootstrapping and Ensemble aggregation turns out to be effective.

**Fig. 2:**
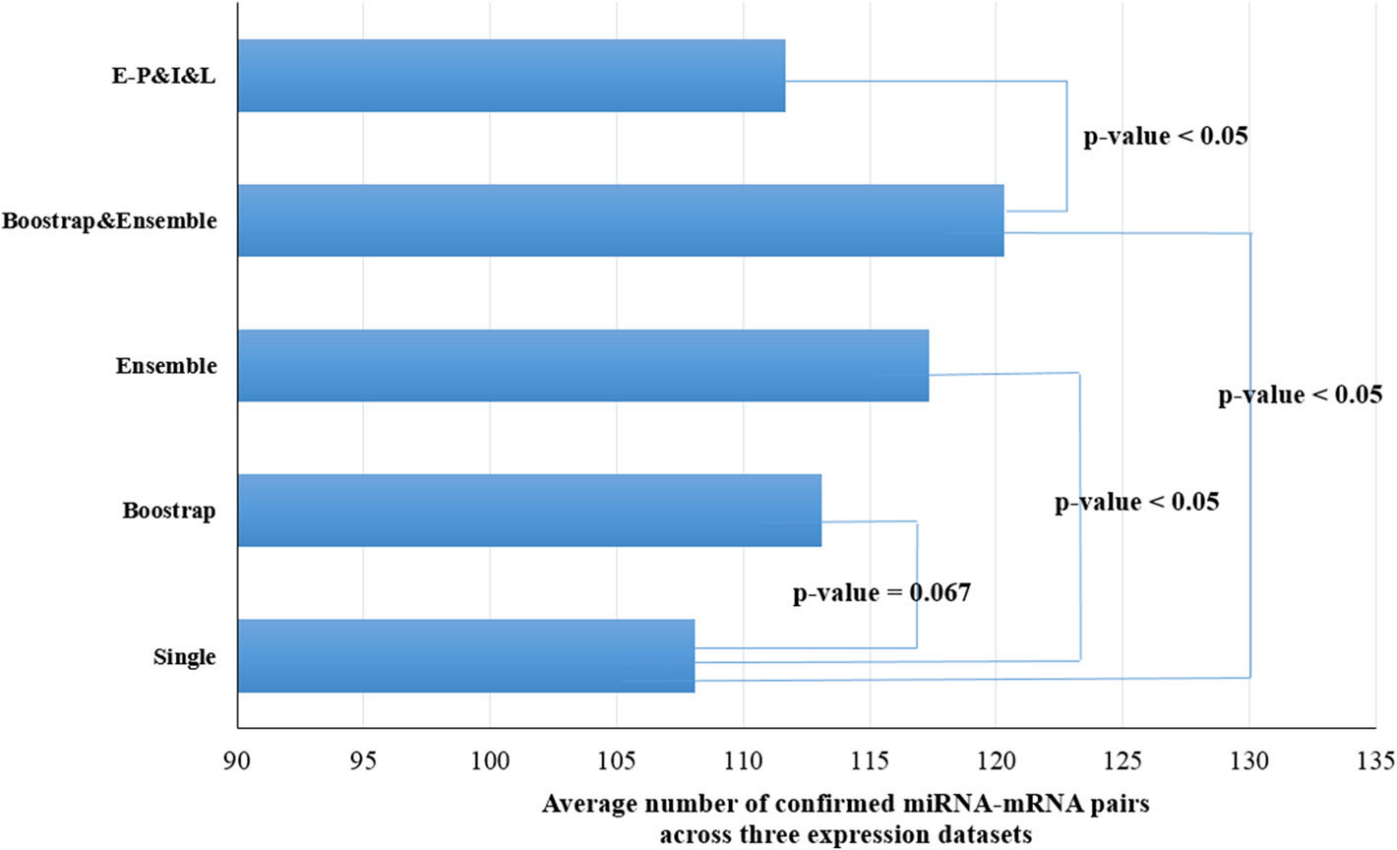
Average number of experimentally confirmed miRNA-mRNA correlations on three datasets. This bar-chart represents the average number of the experimentally confirmed miRNA-mRNA correlations of each method on EMT, MCC and BR datasets. It also shows the statistical significances of differences on performance between two methods in terms of the p-value computed using the paired t-test. ‘Single’ means the average performance of three models from the three direct correlation inference methods without bootstrapping and Ensemble aggregation steps. ‘Bootstrap’ means the average performance of the bootstrapping aggregation results for each three direct correlation inference method. ‘Ensemble’ means the average performance of inferred models using Ensemble aggregation of single experiments. Additionally, Bootstrap&Ensemble means the average performance of proposed DMirNet that uses both bootstrapping and Ensemble aggregation. E-P&I&L means a comparable control that is an ensemble model aggregating Pearson, IDA and Lasso [1]

The effect of bootstrapping and Ensemble approaches can be quantified using a paired t-test. Figure 2 demonstrates the average number of confirmed miRNA-mRNA correlations using each method. Additionally, in order to assess the statistical significance of difference on the precision between two methods, the p-values using the paired t-test were calculated.

We summarize the performance evaluation on precisions for all combinations of DMirNet component using the limited number of ground-truth pairs as follows: 1) The performance of each direct correlation estimation method slightly varies across the three datasets. 2) Applying the bootstrapping procedure generally improves the precision of the model. 3) If an Ensemble model aggregates a poorly performed model, the Ensemble approach guarantees at least the average performance of aggregated methods. 4) The balanced combination of three direct correlation inference methods, bootstrapping and Ensemble approach, strictly reduces the effect of the worst-performed model and achieve the best or the second best precision. Therefore, we demonstrate that the use of both bootstrapping and Ensemble approaches helps to build a more reliable and robust model across different expression datasets, while tackling the large *p* small *n* problem.

### Performance comparison between DMirNet and the state-of-the-art Ensemble-based model

DMirNet framework adopts the three direct correlation network inference methods to identify direct miRNA-mRNA association network. It embeds the bootstrap aggregation for fully utilizing the limited training expression profiles and the Ensemble approaches for improving reliability and performance. To show the effectiveness of DMirNet on identifying direct miRNA-mRNA interactions, we compare the performance of it with the state-of-the-art Ensemble-based model [1]. The Ensemble-based model integrates Pearson’s correlation (denoted as P), IDA (denoted as I) [14], and Lasso (denoted as L) [26] using the Borda count election aggregation method. Through a rigorous comparative study using EMT, MCC, and BR dataset and eight correlation inference methods, the ensemble of P&I&L was selected as a best-performed model across the three datasets [1]. Table 2 shows the number of experimentally confirmed miRNA-mRNA correlations inferred from combinations of components in DMirNet framework and the P&I&L Ensemble model. Table 2 shows interesting results of the comparative study. The solo use of Corpcor, Space, and MIND methods usually does not outperform Pearson, IDA, and Lasso methods. Moreover, Regarding the BR dataset, Pearson, IDA and Lasso rather considerably outperform Corpcor, Space, and MIND with the current ground-truth data. However, when three direct correlation estimation methods are bootstrapped and aggregated, the integrated model considerably performs better. The p-value of the difference on performance between DMirNet (Bootstrap&Ensemble) and P&I&L is less than 0.05 (p-value = 0.040) as shown in Fig. 2. This implies that the difference of the above two methods is statistically significant, and thus, DMirNet is a better choice than P&I&L in a statistical sense.

**Table 2:** Performance comparison of DMirNet with the state-of-the-art Ensemble model

Dataset	Direct correlation inference methods					the state-of-the-art method			
	Corpcor	Space	MIND	E-C&S&M	B&E-C&S&M	Pearson	IDA	Lasso	E-P&I&L
EMT	35	45	24	41	40	30	29	29	31
MCC	200	183	210	209	216	205	198	187	203
BR	98	83	95	102	105	114	124	120	101

To compare the performance of our method with a related work, we investigate the number of experimentally confirmed miRNA-mRNA associations of the state-of-the-art Ensemble model. It combines Pearson’s correlation (denoted as P), IDA (denoted as I), and Lasso (denoted as L) using the Borda count election and was reported as the best-performed Ensemble model on the three datasets [1]. ‘E’ denotes the Ensemble approach, and ‘B&E’ denotes the DMirNet with both bootstrapping and Ensemble aggregation

### Network analysis of inferred direct miRNA-mRNA association networks

Based on the proposed DMirNet framework, we reconstructed direct miRNA-mRNA association networks for each dataset. Through the procedures described in Method section with 100 bootstrapping iterations, the output miRNA-mRNA correlation matrix was generated. We selected top 1000 miRNA-mRNA association relationships to reconstruct association networks for each dataset. The top 1000 miRNA-mRNA pairs for each dataset are listed in the Additional file 1.

**Fig. 3:**
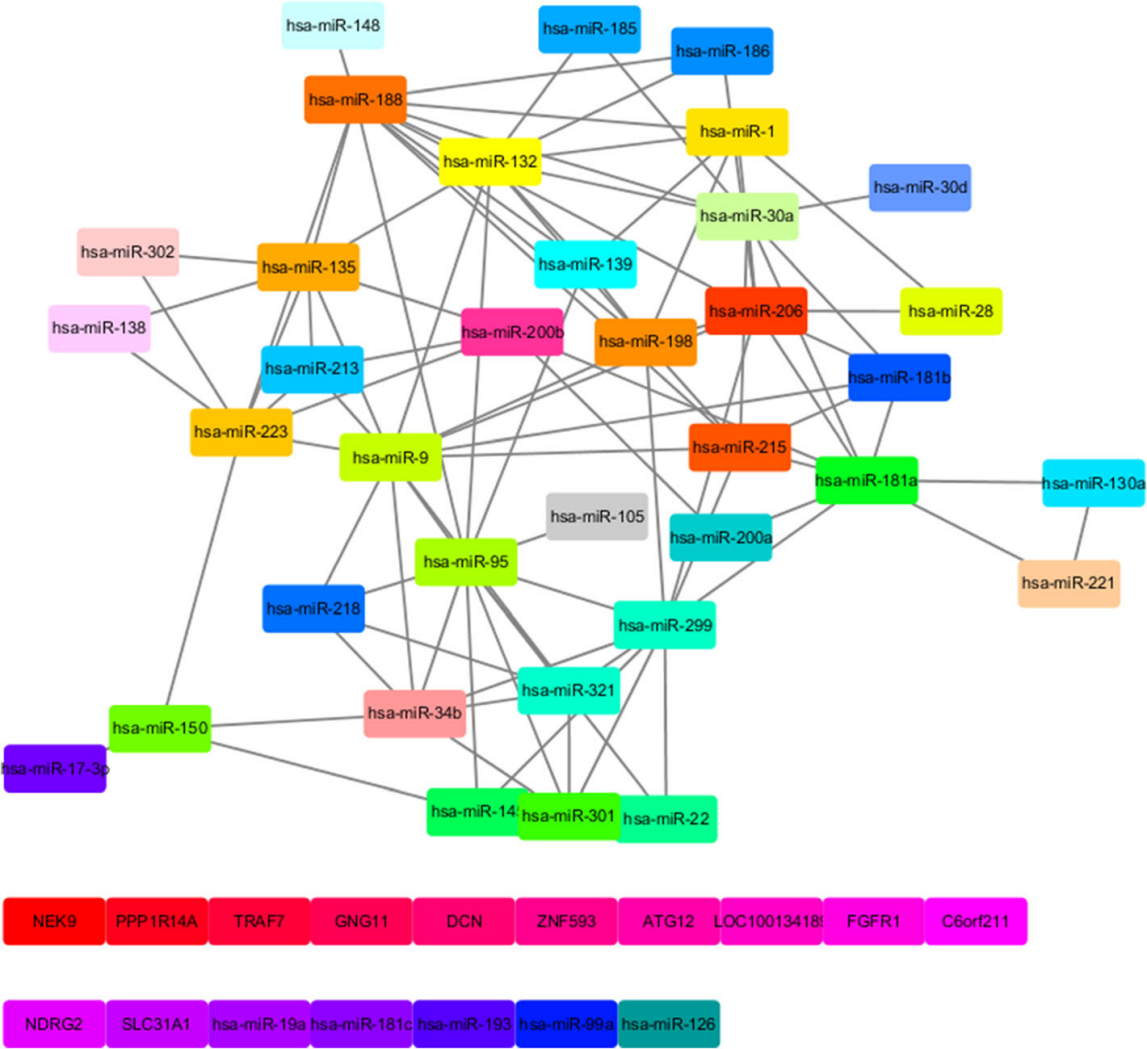
The key network structure of top 500 miRNA-mRNA association network using the MCC dataset. In this key network structure, a node represents a network module, a label of a module represents community centrality, and an edge stands for the connectivity among modules. The network modules identified using ModuLand

We visualized the reconstructed networks from the top 500 pairs using the Cytoscape [41] environment, and analysed their network structure using the ModuLand plug-in [42]. The ModuLand can determine overlapping network modules and community centrality. Since the outputs of the ModuLand represent representative community centralities and connections among network modules, the results of the ModuLand effectively show an abstraction of the whole network structure. For each dataset, the reconstructed network and its key network structure are shown in the Additional file 2. Among them, Fig. 3 shows the core network structure of the inferred Top 500 miRNA-mRNA associations of the MCC data. Additionally, the network of modules represents the indirect associations among miRNAs mediated by the mRNAs.

**Fig. 4:**
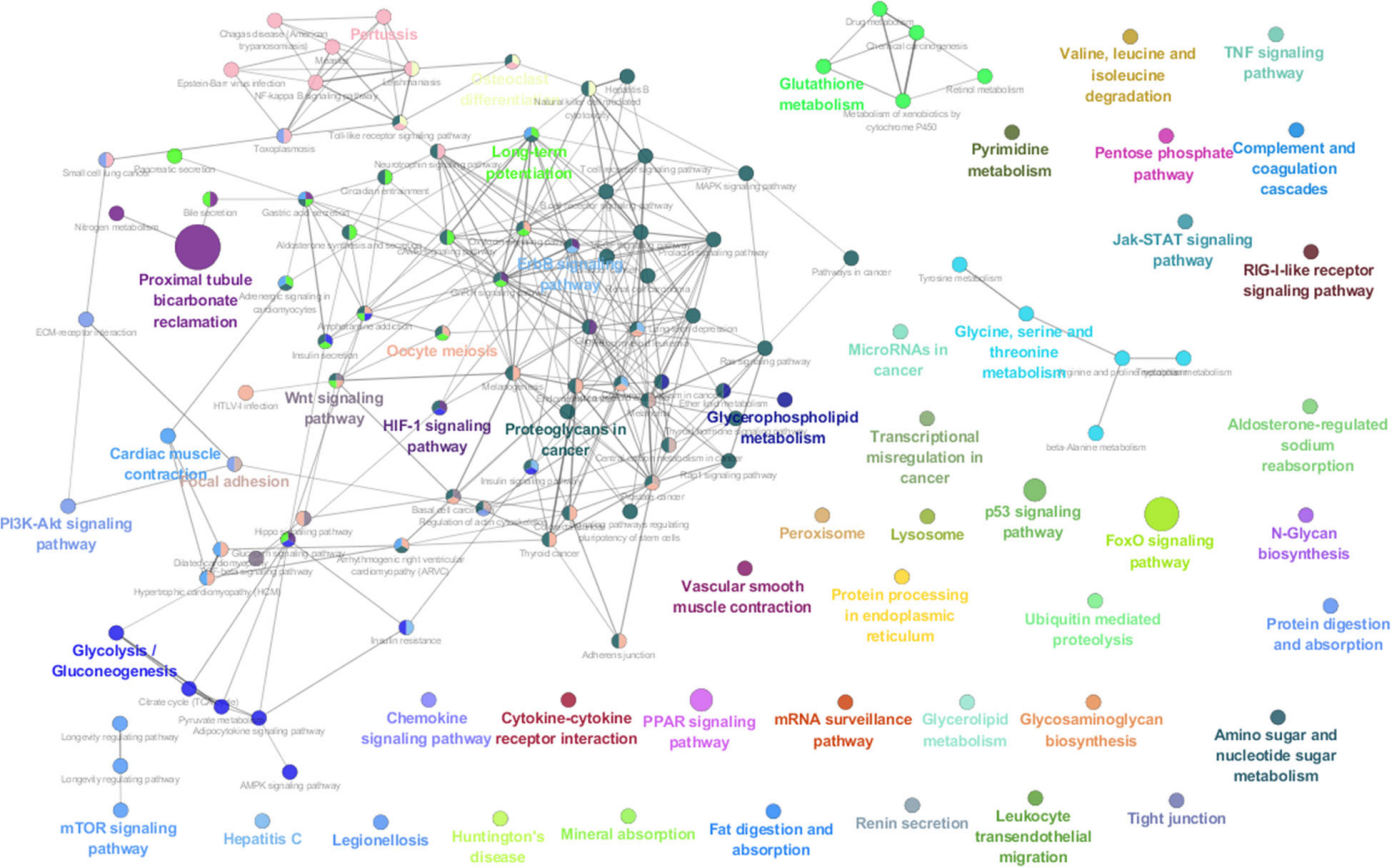
KEGG biological pathways related to Top 1000 pairs of the MCC dataset

**Fig. 5:**
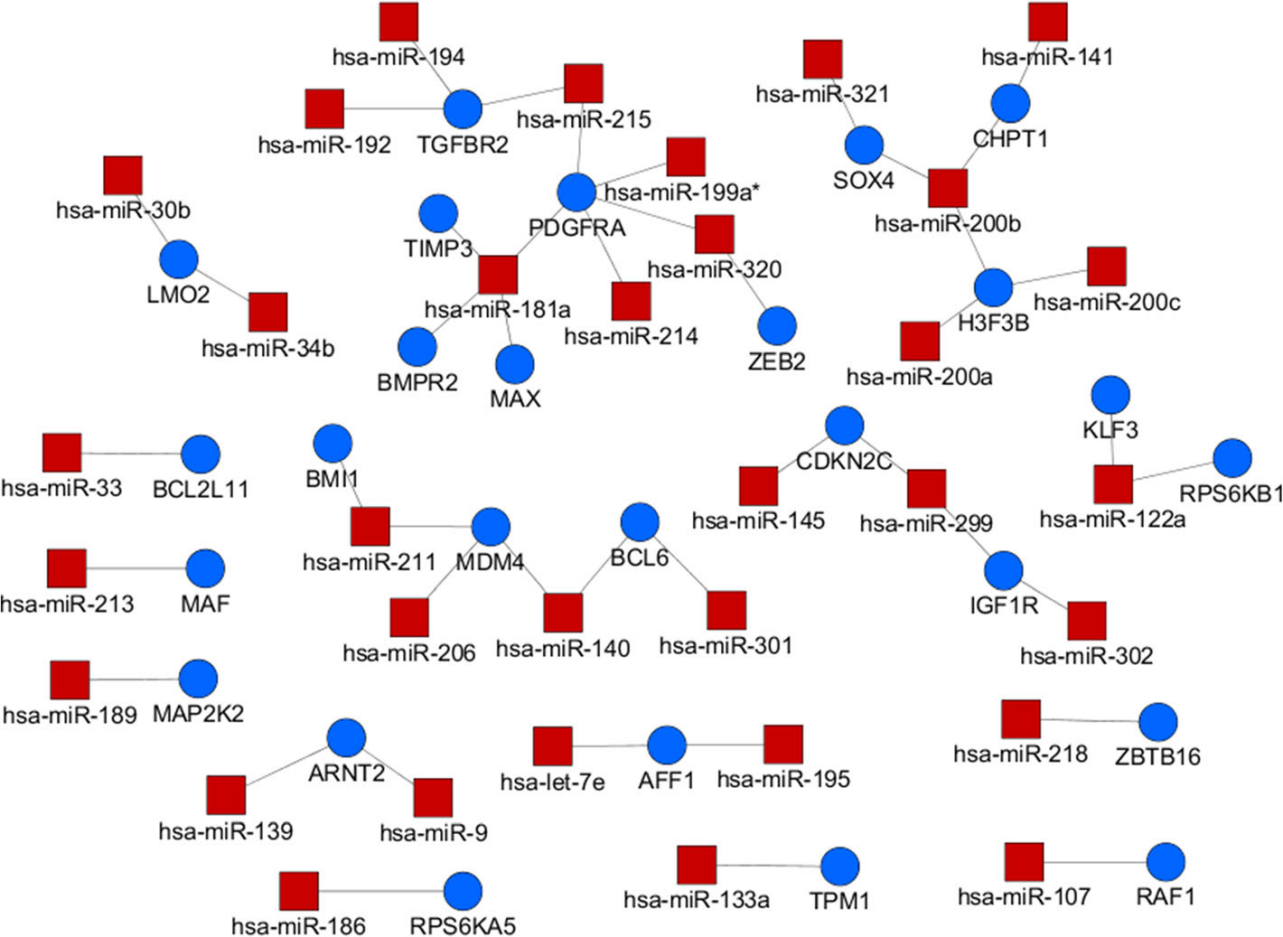
Cancer-related miRNA-mRNA association networks among Top 1000 pairs of the MCC dataset. The red rectangle nodes are mRNAs and the blue circle nodes are mRNAs

To interpret the related biological pathway of inferred miRNA-mRNA association network, we analyse the functions of mRNAs listed in the Top 500 and Top 1000 pairs based on KEGG pathway [43]. We used the ClueGO [44] Cytoscape plug-in to extract the biological pathways for associated mRNAs, and to visualize the selected KEGG pathway terms in a functionally grouped network. The overall results of identifying significant KEGG pathway across three dataset are summarized in the Additional file 3. Figure 4 demonstrates the KEGG biological pathways related to Top 1000 pairs of the MCC dataset. The size of the nodes reflects the statistical significance of the terms. The degree of connectivity between terms (edges) is calculated using kappa statistics. The calculated kappa score is also used to define functional groups. A node having more than two colours is a term that can be included in several groups. It should be noted that the MCC dataset is the expression profiles of normal and cancerous tissues from eight organs. The top 1000 pairs of the MCC dataset consists of 103 miRNAs and 572 mRNAs. The biological pathway analysis was performed on the 572 mRNAs. Among various biological pathways, there are three cancer-related categories; namely ‘Transcriptional misregulation in cancer,’ ‘MicroRNAs in cancer,’ and ‘Choline metabolism in cancer.’ The three cancer-related categories are associated with 44 miRNA-mRNA pairs among 33 miRNAs and 27 mRNAs. The list of the 44 miRNA-mRNA pairs is shown in Additional file 4, and Fig. 5 shows the list in a network form.

To investigate putative novel multi-cancer-related miRNA-mRNA pairs, we checked the overlaps between the 44 multi-cancer-related miRNA-mRNA pairs and ground-truth data which is a union of the four manually curated database. Our DMirNet found out a strong miRNA-mRNA association between hsa-miR-181a and BMPR2 as top 809 out of 200,880 pairs (upper 0.4% percentile). This miRNA-mRNA relationship has already been confirmed in [45] such that the hsa-miR-181a plays a direct role in down-regulating the BMPR2. This means that our DMirNet inference provide a consistent result with pre-known miRNA-mRNA relationships.

Regarding hsa-miR-299::CDKN2C (top 479) and hsa-miR-301::BCL6 (top 593) in the 44 multi-cancer-related pairs, they are not listed in the ground-truth data. However, the ground-truth data includes closely related pairs (namely, has-miR-299-5p::CDKN1A and hsa-miR301a::BCL2L11) of which mRNA is from the same gene family. In many cases, genes in a family have a similar structure of function, or proteins produced from these genes work together as a unit or participate in the same process. Therefore, the existence of similar miRNA-mRNA pair may support the plausibility of the inferred pairs by DMirNet.

After excluding the known miRNA-mRNA pair (hsa-miR-181a::BMPR2), 43 among 44 miRNA-mRNA pairs can accordingly be regarded as the putative novel multi-cancer-related miRNA-mRNA pairs.

## Discussion

By investigating the combinatorial effect of the bootstrapping and the Ensemble aggregation on DMirNet framework, the performance enhancement factors of DMirNet are demonstrated. The bootstrapping procedure helps to build a more accurate and reproducible network by fully utilizing the limited size of samples. Additionally, the Ensemble model helps to avoid the worst performance by guaranteeing at least the average performance of aggregated methods. The balanced combination of three direct correlation inference methods, bootstrapping and Ensemble approach, strictly reduces the effect of the worst-performed model and achieves a better precision.

Additionally, when we compare the performance of DMirNet with P&I&L, three single direct correlation inference methods do not show good performance compared to Pearson, IDA, and Lasso. This result indicates that even though each direct correlation estimation method suppresses its indirect information from an observed data in some degree, they are still incomplete. However, by incorporating the bootstrapping and Ensemble aggregation, DMirNet outperforms the best-performed P&I&L across three datasets. These results demonstrate the effectiveness of DMirNet procedure in terms of accuracy and robustness. Although the three direct correlation inference methods cannot perfectly suppress the whole indirect relationships from the observed data, we can effectively focus on the direct associations through incorporating the bootstrapping and the Ensemble approach. We expect that if we can integrate more direct correlation inference methods to DMirNet, the performance of DMirNet would be more improved. Also, if Pearson, IDA, and Lasso methods can be integrated with additional information such as sequence-based miRNA-mRNA target prediction result, the indirect associations might be filtered, and it may further improve the performance of the Ensemble model.

We would like to discuss the limitation of the ground-truth dataset which was used at the experiments. The number of pairs in ground-truth data is significantly smaller than the expected number of miRNA-mRNA correlation pairs in a genome. Moreover, the miRNA-mRNA relationships are dynamically changed according to the experimental method, sample, and experimental condition. For example, ‘hsa-miR-19a-3p’ sometimes directly down-regulates the mRNA of RAB14 on the Kidney tissue with the PAR-CLIP experiment [46], whereas ‘hsa-miR-19a-3p’ sometimes does not regulate RAB14 on the lung tissue with the Luciferase reporter assay method results [47] as shown in Table 3. Therefore, it is difficult to fully estimate the performance of computational inference methods based only on the overlap with the limited size of the ground-truth data. We expect that the number of experimentally confirmed pairs will increase as miRNA mediated gene regulation research in this field becomes more mature and flourished. More extensive ground-truth findings may confirm our false negative inference cases as true positive ones.

**Table 3:** Conflict between experimental results on hsa-miR-19a-3p and RAB14

Publication	Method	Tissue	Cell line	Tested cell line	Result	Regulation
Hafner M. et al. 2010 [46]	PAR-CLIP	Kidney	HEK293	N/A	Positive	Down
Kanzaki H et al. 2011 [47]	Luciferase Reporter Assay	Lung	SBC3	HEK293	Negative	?

A manually curated miRNA-target database includes conflict experimental results for some miRNA-mRNA pairs. As an example, this table shows a conflict experimental result on hsa-miR-19a-3p and RAB14(hsa) from TarBase 6.0 [37]

Regarding the MCC datasets, we identify putative novel multi-cancer-related miRNA-mRNA pairs by utilizing KEGG pathway analysis and ground-truth data. After excluding previously known one pair and similar two pairs with the ground-truth data, 43 out of 44 miRNA-mRNA association pairs are reported.

Although our DMirNet improves the performance by incorporating the bootstrapping and Ensemble approach, the bootstrapping procedure may come with computational overhead. The bootstrapping procedure generates *m* training datasets using sampling with replacement, computes *m* direct correlation matrices, and aggregates the *m* models. If the bootstrapping procedures are combined with Ensemble approach that aggregates *n* different methods, we have to run the bootstrapping procedure *n* times. However, in many bioinformatics applications, there is a trade-off between performance improvement and computation complexity. Also, we can accelerate the bootstrapping and ensemble procedure by utilizing the MPI.

## Conclusions

We have presented the DMirNet framework that identifies direct miRNA-mRNA association networks from expression profiles. DMirNet takes full advantage of three direct association estimation methods, the bootstrapping and the Ensemble approach based on an inverse-rank-product method. The performance evaluation has shown a substantial effectiveness of DMirNet in terms of the number of the matched miRNA-mRNA cases with a ground-truth data. Our proposed DMirNet framework outperforms the state-of-the-art Ensemble model with a factor of up to 1.29 with the EMT data in terms of precision. These empirical experimental results show the effectiveness of the combinatorial effects of the direct association estimation, the bootstrapping, and the Ensemble approaches in DMirNet. This paper demonstrates that our DMirNet can be a promising alternative to other existing methods to identify direct and novel miRNA-mRNA relationships more extensively. We expect that DMirNet can contribute to reconstructing various direct regulatory pathways, including, but not limited to, the direct miRNA-mRNA association networks.

## Additional files

Additional file 1: Top1000 miRNA-mRNA association relationships for each dataset. (XLSX 59 kb)
Additional file 2: Reconstructed miRNA-mRNA association networks and their key structures with top 500 miRNA-mRNA association relationships. The reconstructed network was visualized with Cytoscape and the key modular structure of the network was analysed using ModuLand. (XLSX 658 kb)
Additional file 3: Functional analysis of reconstructed miRNA-mRNA association networks based on KEGG pathway. To interpret the functions of inferred miRNA-mRNA association network, related KEGG pathway in Top 500 and Top 1000 pairs were analysed using ClueGO (XLSX 1447 kb)
Additional file 4: The list of multi-cancer-related 44 miRNA-mRNA pairs. The putative novel multi-cancer-related pairs are coloured with yellow. (XLSX 12 kb)
